# Fleming Fund Implementation in Kenya: What Have We Learned About Strengthening One Health Antimicrobial Resistance Surveillance for Decision-making?

**DOI:** 10.1093/cid/ciag500

**Published:** 2026-10-19

**Authors:** Evelyn Wesangula, Nkatha Gitonga, Emmanuel Tanui, Romona Ndanyi, Susan Githii, Bridgit Syombua Muasa, Eunice Omondi, Eric M Fèvre, Dishon M Muloi, Ali Kassim, Josiah Mwenda, Christine Minoo Mbindyo, Irungu John Kamau, Loice Achieng Ombajo, Gunturu Revathi, Veronica Chuchu, Anafi Mataka, Moctar M M Mouiche, Arshnee Moodley, Stanley Fenwick, Deborah Nanjebe, Lillian Bibangamba, Patrick Mubangizi, Toby Leslie, John A Crump, Zulfiqar Ahmed Bhutta, Tim Walsh, Kate Macintyre, Rebecca Maudling, Solomon Mugabe, Raheelah Ahmad, Lamiaa Shehata, Tim Shorten

**Affiliations:** Family Health and Infectious Diseases, East Central and Southern Africa Health Community, Arusha, Tanzania; Itad, United Kingdom; Global Health—AMR, Nairobi, Kenya; Division of Quality Improvement, Infection Prevention and Control and Antimicrobial Resistance, Kenya National Public Health Institute, Nairobi, Kenya; State Department of Livestock Development, Directorate of Veterinary Services, Nairobi, Kenya; National Laboratory Science and Biosecurity, Kenya National Public Health Institute, Nairobi, Kenya; State Department of Livestock Development, Directorate of Veterinary Services, Nairobi, Kenya; State Department of Livestock Development, Directorate of Veterinary Services, Nairobi, Kenya; International Livestock Research Institute (ILRI), Nairobi Kenya; Institute of Infection, Veterinary and Ecological Sciences, University of Liverpool, Liverpool, United Kingdom; International Livestock Research Institute (ILRI), Nairobi Kenya; Department of Laboratory Medicine, Kenyatta National Hospital, Nairobi, Kenya; Department of Veterinary Pathology, Microbiology and Parasitology, Faculty of Veterinary Medicine, University of Nairobi, Nairobi, Kenya; Department of Veterinary Pathology, Microbiology and Parasitology, Faculty of Veterinary Medicine, University of Nairobi, Nairobi, Kenya; Division of Quality Improvement, Infection Prevention and Control and Antimicrobial Resistance, Kenya National Public Health Institute, Nairobi, Kenya; Department of Medicine, University of Nairobi, Nairobi, Kenya; Aga Khan University Hospital, Nairobi, Kenya; Commonwealth Pharmacists Association, London, United Kingdom; African Society for Laboratory Medicine, Addis Ababa, Ethiopia; African Society for Laboratory Medicine, Addis Ababa, Ethiopia; International Livestock Research Institute (ILRI), Nairobi Kenya; University of Copenhagen, Copenhagen, Denmark; Mott MacDonald; Mott MacDonald; Mott MacDonald; Mott MacDonald; Mott MacDonald; Otago Global Health Institute, University of Otago, Aotearoa, New Zealand; The Aga Khan University Centre for Global Child Health; Fleming Fund Technical Advisory Group AG; University of Oxford; Itad, United Kingdom; Oxford Health Evaluations, United Kingdom; Itad, United Kingdom; Independent Consultant, Brighton, United Kingdom; Mott MacDonald; City St George's, University of London; Fleming Initiative, Imperial College, London, United Kingdom; Itad, United Kingdom; Itad, United Kingdom; Independent Consultant, Brighton, United Kingdom

**Keywords:** antimicrobial resistance, One Health, surveillance, laboratory strengthening, governance

## Abstract

**Background:**

Kenya has moved from fragmented, pilot-based antimicrobial resistance (AMR) surveillance toward a coordinated One Health system, and its experience, pathways of change, and remaining gaps are relevant for other low- and middle-income settings working to operationalize One Health AMR surveillance.

**Methods:**

Here, we report a narrative overview of programmatic learning using 2018 as the pre-Fleming Fund baseline, drawing on national and global surveillance reports, monitoring frameworks from the Quadripartite indicators, qualitative evidence on governance, implementation and policy evolution across the One Health sectors, and Fleming Fund documentation.

**Results:**

Since 2018, AMR surveillance expanded from 2 human health sites to 30 human, animal, and environmental sites by 2025. National reports evolved to integrate AMR, antimicrobial consumption, and antimicrobial use data; routine reporting to global platforms matured; and outputs such as dashboards, reports, and policy briefs informing stewardship, Infection Prevention and Control, regulatory action, and policy decisions. External benchmarks showed improvement in AMR-related capacity, with World Health Organization Joint External Evaluation scores moving from limited to demonstrated capacity for surveillance and multisectoral coordination.

**Conclusions:**

Kenya's experience suggests that sustainable progress depends on embedding AMR surveillance within government-led governance structures, technical systems, and coordinated partner support, but sustaining gains will require predictable financing and routine data use.

This article is part of a journal series synthesizing evidence and lessons from the Fleming Fund to contribute to wider learning on how complex global health programs translate technical capacity into institutional and policy change and to strengthen structured programmatic learning within UK-funded antimicrobial resistance (AMR) programming and the wider literature.

Antimicrobial resistance is a growing threat to global health security, food systems, and sustainable development, with severe effects in low- and middle-income countries (LMICs), where infectious disease burdens are high and health systems are variably resourced [1, 2]. Antimicrobial resistance is increasingly understood as a One Health challenge spanning human, animal, and environmental health, as water systems, wastewater, and environmental contamination serve as reservoirs and transmission pathways for resistant organisms [3]. Bacterial AMR contributed to an estimated 1.14 million deaths globally in 2021, with sub-Saharan Africa bearing a disproportionate burden [4]. Recent surveillance data indicate that approximately 1 in 6 bacterial infections reported in 2023 were resistant to commonly used antibiotics [5].

In Kenya, AMR is characterized by high resistance levels. Among endemic enteric infections linked to environmental transmission pathways, only 18% of *Salmonella typhi* isolates are fully susceptible to commonly available antimicrobials, while 60%–68% are multidrug resistant, illustrating how environmental contamination and poor water and sanitation infrastructure drive resistance [6]. These patterns are driven by linked human, animal, and environmental factors, reinforcing the need for surveillance-driven, multisectoral action [6, 7]. In response, Kenya was among the first sub-Saharan African countries to prioritize tackling AMR through a multisectoral approach [8]. Early situational analyses in 2011 and 2016 identified resistant pathogens across human and animal sectors alongside fragmented laboratory capacity, surveillance, and data use, directly informing the establishment of the National Antimicrobial Stewardship Interagency Committee (NASIC) in 2014 followed by the 2017 National Policy on AMR and the first National Action Plan (NAP-AMR 2017–2022) [8–12]. While Kenya had already institutionalized a broader One Health approach in 2011 through the Zoonotic Disease Unit, NASIC provided the dedicated multisectoral coordination architecture required for AMR (Figure 1) [13].

**Figure 1.**
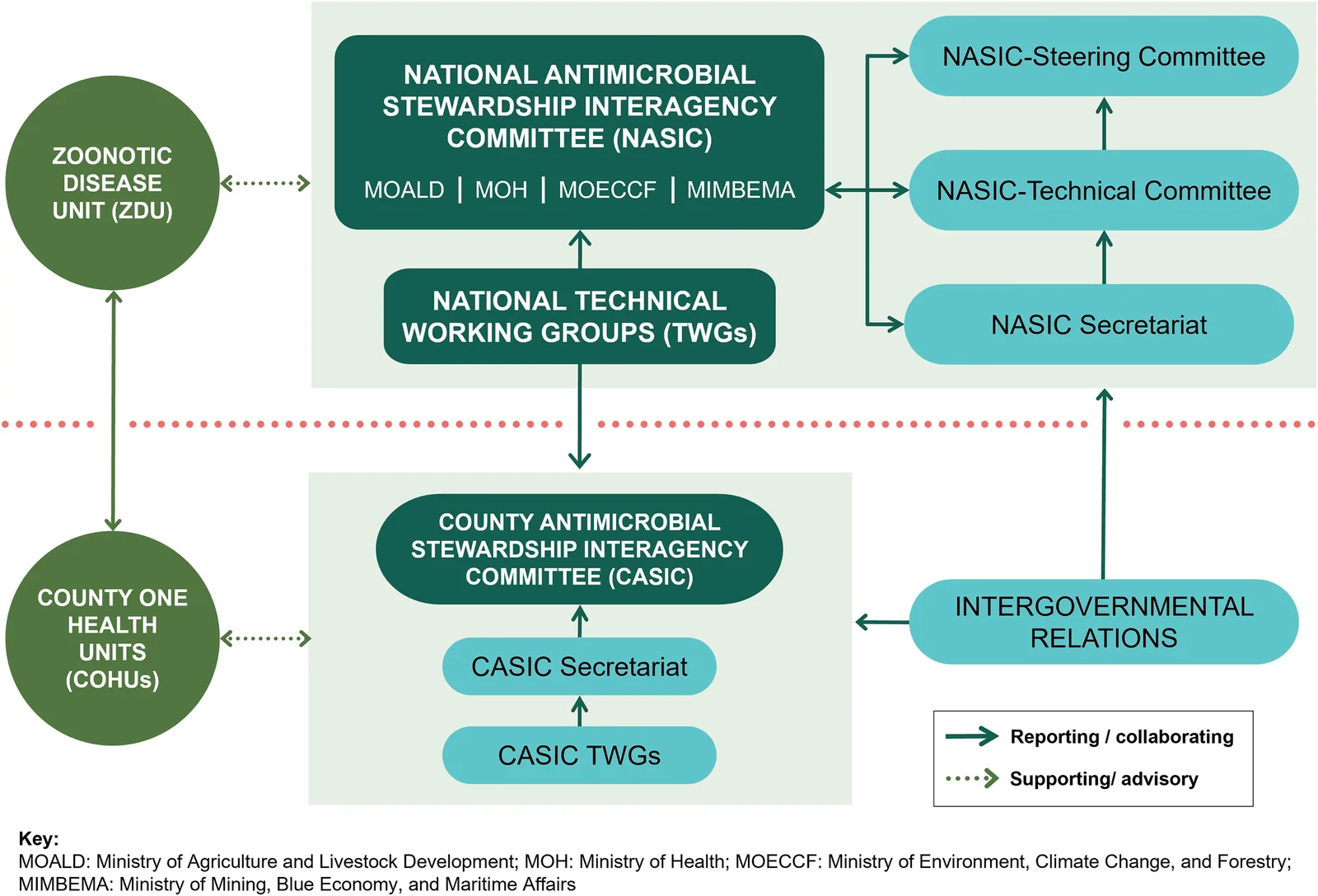
Antimicrobial resistance (AMR) coordination mechanism in Kenya showing national and subnational (county) structures.

Surveillance is central to AMR containment efforts, providing the intelligence needed to guide stewardship, infection prevention and control (IPC), and policy action. While international frameworks provide overarching guidance for AMR containment, and global monitoring platforms such as World Health Organization (WHO) Global Antimicrobial Resistance and Use Surveillance System (GLASS), the World Organization for Animal Health (WOAH) Animal Antimicrobial Use (ANIMUSE), and the Food and Agriculture Organization (FAO) AMR Monitoring (InFARM) standardize data reporting across sectors, there is limited published evidence on how these global standards and tools are operationalized in LMIC settings, particularly in devolved systems such as Kenya where national and subnational coordination must coexist [14–16]. This paper examines how Kenya's One Health AMR surveillance system evolved between 2018 and 2025, using a narrative programmatic learning synthesis to identify the factors that enabled or constrained progress and to develop transferable implementation principles for comparable settings.

## EVIDENCE, APPROACH, AND METHODS

### Study Design and Analytical Framework

This paper forms part of a practice-informed series synthesizing learning from Fleming Fund-supported activities. The companion papers include One Health implementation, data production and use, and a forward-looking paper reflecting on future surveillance systems [17–20]. It draws on program documentation, evaluation evidence, relevant literature and structured engagement with a broad range of contributors from multiple disciplines and perspectives, including the National AMR Coordinating Committee, Fleming Fund implementing partners, and the wider AMR community in Kenya.

This paper uses a narrative programmatic learning synthesis to describe how Kenya's AMR surveillance system evolved between 2018 (pre-Fleming Fund baseline) and 2025. The analysis draws on a descriptive policy implementation framework, focusing on how governance structures, technical systems, and data use routines changed over time and how external support interacted with national priorities and existing institutions. The unit of analysis is the national One Health surveillance system, encompassing human, animal, and environmental sectors. The paper does not constitute an independent evaluation or seek to establish causal attribution. Findings should therefore be interpreted in light of limitations including program-linked authorship, contributor perspectives and purposive source selection.

### Data and Information Sources

National AMR surveillance data and reports were reviewed to assess changes in data generation, analysis, and reporting since 2018. Sources included annual national AMR surveillance reports and technical outputs from human, animal, and environmental sectors. The review focused on One Health-relevant indicators of surveillance maturity, including expansion of surveillance sites, pathogens reported, antimicrobial susceptibility testing (AST) completeness, consistency of reporting to national and international platforms, use of antibiograms, and growing incorporation of antimicrobial consumption (AMC) and antimicrobial use (AMU) data using standardized methods. Documentation from AMR and antimicrobial stewardship (AMS) technical working groups (TWGs) supplemented this review by capturing coordination processes, stewardship guidance development, and the use of surveillance outputs to inform actions across sectors.

Fleming Fund documentation, including Kenya Country Grant phase 1 and 2, relevant Regional Grants, and fellowship documentation, was reviewed to assess investments, implementation approaches, and support for laboratory quality, data harmonization, AMC and AMU analysis, and workforce development. Data submitted by Kenya to WHO GLASS, WOAH ANIMUSE, and FAO InFARM, together with the 2017 and 2024 WHO Joint External Evaluations (JEE) and selected Tracking AMR Country Self-Assessment Survey indicators, were used to triangulate findings across self-reported and externally assessed capacity.

### Methodological Limitations

This is a narrative synthesis, not a systematic review, relying on published reports, program documentation, and secondary data. Data completeness and comparability may vary across counties and sectors, particularly for the environmental sector where surveillance was introduced later. Attributing specific outcomes solely to Fleming Fund investments is constrained by the multipartner ecosystem in which implementation occurred. Qualitative observations on governance and policy evolution are drawn from program documentation and stakeholder accounts, which may reflect reporting and documentation and recall bias.

## WHAT FLEMING FUND SUPPORTED IN KENYA

Since 2018, the Fleming Fund has supported One Health country grants, regional grants, strategic alignment grants, and fellowship schemes as catalytic investments aligned to national AMR policies and plans [21–23]. These initiatives strengthened laboratories, pharmacy, veterinary and environmental surveillance systems, workforce capacity, data platforms, and multisectoral coordination to advance integrated, decision-oriented surveillance.

### Implementation Approach and Governance Arrangements

Kenya's implementation model was government-led, involving the ministries responsible for health, agriculture and livestock, and environment and their agencies, regulatory bodies, academic partners, and implementation partners through phased grants that allowed early diagnostic and data generation work in human and animal health to inform subsequent investments in governance, data integration, and sustainability.

### Core Investment Areas

Fleming Fund support in Kenya, in line with the country Request for Proposal, was organized around 4 core, cross-sector investment areas (Table 1) that were deliberately embedded in existing national systems rather than run as parallel projects [23].

**Table 1.** Core Investment Areas Funded by the Fleming Fund in Kenya

Core Investment Area	One Health Scope (Human/Animal/Environment)	Main Fleming Fund–Supported Actions and Alignment With National Systems
1) Laboratory capacity and quality management	Human, animal, and environmental laboratories	Supported selected hospital, veterinary, and environmental laboratories through refurbishment, equipment, consumables, and standardized culture and AST systems. Investments were aligned to the National AMR Surveillance Strategy 2018–2022 and the One Health Implementation Plan 2023–2027 [24, 25]. Human and animal laboratories were linked through reference laboratory and hub-and-spoke networks, respectively. Quality management systems strengthening aligned to ISO 15189 and ISO/IEC 17025:2017, alongside participation in national, regional, and EquAfrica external quality assurance (EQA) schemes.
2) Workforce development and mentorship	Multidisciplinary human, animal, and environmental workforce	Built competencies in microbiology, epidemiology, sampling, data handling and analysis, diagnostic stewardship, and AMR leadership among human health, animal health and environmental professionals. Support included mentorship frameworks, surveillance protocols across sectors, farm biosecurity and prudent AMU guidance, and institutionalized continuous professional development platforms such as ECHO TEACH AMS and the MoH e-Learning Academy [26].
3) Surveillance and data systems	Integrated AMR, AMU, and AMC across sectors	Expanded surveillance sites, standardized tools, and strengthened data flows into the AMR Central Data Warehouse (CDW). These investments supported reporting to GLASS, ANIMUSE, and InFARM, while generating national AMR annual AMR reports [7, 27–29], dashboards [30, 31], trend analyses, and briefs that inform AMS, IPC, biosecurity, and procurement decisions. In-country sequencing of human, animal, and environmental samples strengthened genomics and bioinformatics capacity and improved characterization of resistance pathogens.
4) One Health coordination mechanisms—national–county interface	Governance across sectors and levels of government	Reinforced NASIC, CASICs, and TWGs as platforms for reviewing surveillance data, validating analyses, and translating findings into national and county actions, including stewardship guidance, veterinary prescribing norms, planning, and budgeting.

Abbreviations: AST, antimicrobial susceptibility testing; AMR, antimicrobial resistance; AMU, antimicrobial use; AMC, antimicrobial consumption; NASIC, National Antimicrobial Stewardship Interagency Committee; CASIC, County Antimicrobial Stewardship Interagency Committee; TWG, technical working group; IPC, infection prevention and control; AMS, antimicrobial stewardship; ANIMUSE, Animal Antimicrobial Use; GLASS, Global Antimicrobial Resistance and Use Surveillance System.

### Alignment With Existing National Policies and Systems

The Fleming Fund support was designed as a catalytic addition to Kenya's existing AMR framework, aligning grants, fellowships, and technical assistance with national policies, sector plans, and county structures, through the single country investment strategy. Coordination was anchored through NASIC, County Antimicrobial Stewardship Interagency Committees (CASICs), and the multisectoral TWGs as the primary coordination mechanisms. Figure 2 shows how these inputs translated into outputs, outcomes, and impact.

**Figure 2.**
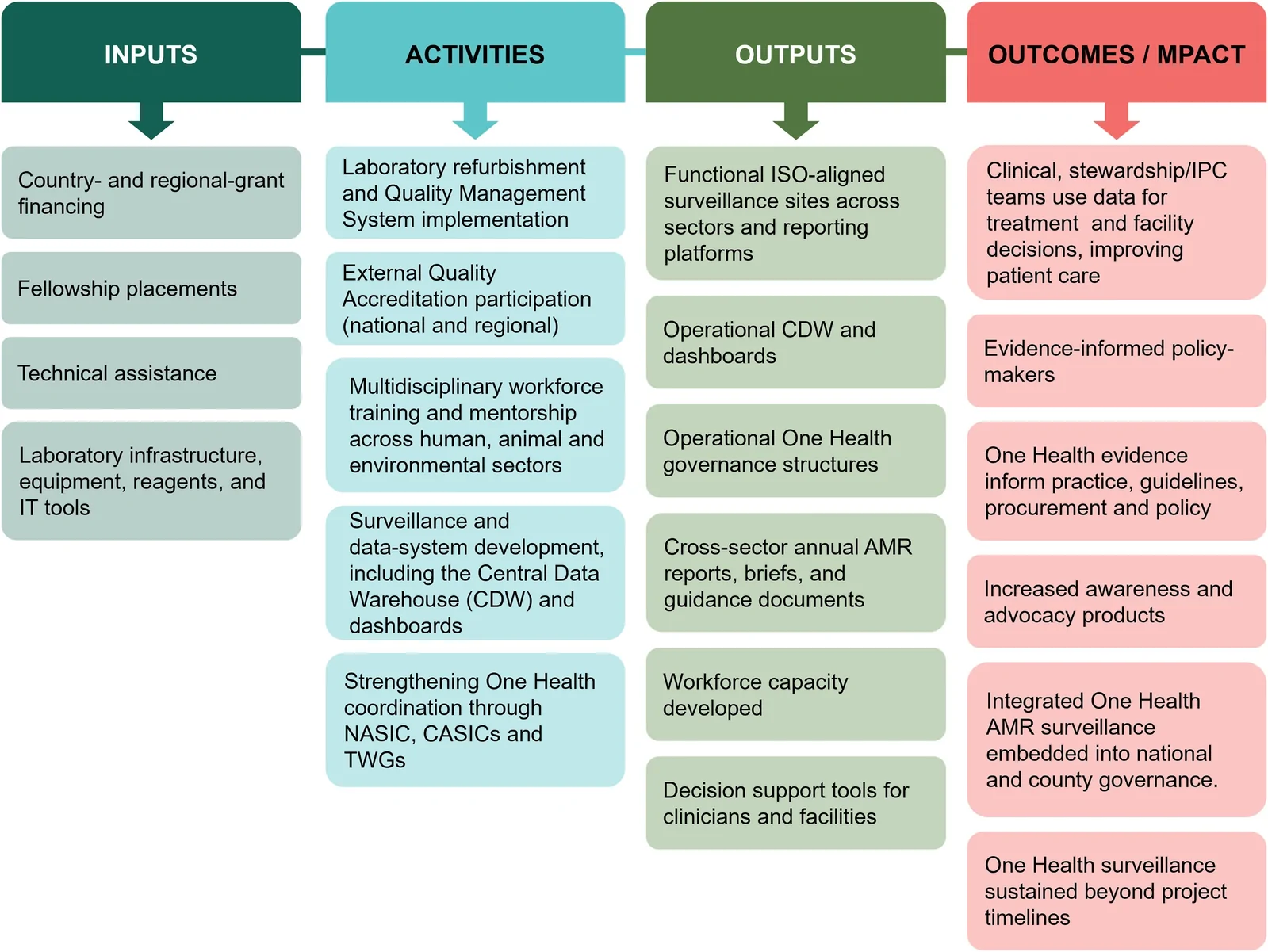
Results-chain diagram showing how Fleming Fund inputs, activities, and outputs feed into the outcomes/impact.

By anchoring every investment to existing legal and strategic instruments and delivering concrete outputs such as policy briefs, the Fleming Fund enabled Kenya decision-makers to translate surveillance data into actionable national policy, county, and facility-level plans, including clinician-level decision-making.

## RESULTS: WHAT CHANGED SINCE 2018?

Kenya has made measurable progress in strengthening AMR surveillance and governance across human, animal, and environmental sectors. Compared with the fragmented pre-2018 baseline, the country now demonstrates stronger governance, expanded laboratory and surveillance platforms, and growing use of AMR, AMC, and AMU data to inform practice and policy.

### Governance and One Health Coordination Under Devolution

Kenya's AMR governance architecture strengthened as NASIC evolved from an advisory forum into an inclusive routine decision-making platform, while multisectoral TWGs began reviewing surveillance data, validating annual reports, and informing policy, guidelines, formularies, diagnostic priorities, and IPC/AMS workstreams. Under Kenya's devolved system, CASICs expanded from 4 counties in 2018 to 22 by the end of 2025, providing formal mechanisms for oversight, resource allocation, and national feedback [7].

Key governance advances include the following:

- Bidirectional focal-point linkage: County AMR focal points participate in national review forums, enabling exchange of surveillance trends and implementation challenges.
- Joint surveillance data review: Counties present AMR-related data for validation by the national TWGs.
- Multisectoral report validation: Human, animal, environment, regulatory, and academic partners jointly endorse reports and ensure methodological consistency.
- Anchoring AMR and IPC within Kenya National Public Health Institute reflects maturation from program-based implementation to institution-based governance structures responsible for surveillance, preparedness, laboratory strengthening, and public health workforce development as a sustainable platform for action beyond donor cycles.

### One Health Laboratory Capacity and Quality Systems

Kenya moved from fragmented, pilot-based AMR surveillance in 2018 to a more integrated One Health platform by 2025, with expansion across all 3 sectors in laboratory coverage, sample volumes, quality systems, and sectoral inclusion (Table 2).

**Table 2.** Comparative Summary of One Health AMR Surveillance Coverage, 2018 Versus 2025

Indicator	2018 Baseline	2024–2025 Status
Human health surveillance sites	2 sites in 2 counties	20 sites in 15 counties (2024)
Human samples in central data warehouse	Approximately 64 per year	20 299 (2024)
Animal health surveillance	Passive in situ testing in 7 laboratories	Passive and active surveillance through a 9-site hub-and-spoke network
Animal samples in central data warehouse	Not systematically recorded	5425 (2024), up from 2106 in 2022
Environmental surveillance	Absent at baseline	National sentinel site established; 58 ESBL-producing isolates by end 2024
External quality assurance acceptable scores	13/26 (50%)	23/28 (82%)
Reagent and consumable stock-outs	14 (2023)	1 (2025)
Reporting to global platforms	Not routinely achieved	Routine reporting to WHO, GLASS, WOAH, ANIMUSE, and FAO InFARM

Abbreviations: ESBL, extended-spectrum beta-lactamase; WHO, World Health Organization; GLASS, Global Antimicrobial Resistance and Use Surveillance System; WOAH, World Organization for Animal Health; ANIMUSE, Animal Antimicrobial Use; FAO, Food and Agriculture Organization.

In human health, surveillance expanded from 2 sites in 2 counties in 2018 to 20 sites in 15 counties by the end of 2024 [7, 27], while annual human samples processed and recorded in the central data warehouse increased from approximately 64 to 20 299 (Figure 3). In animal health, surveillance evolved from passive in situ testing in 7 laboratories to a 9-site hub-and-spoke network, reflecting a shift toward active surveillance. Animal samples recorded in the central data warehouse increased from 2106 in 2022 to 5425 in 2024, a 91% increase driven by enhanced active surveillance activities with the support of the Fleming Fund (Figure 4).

**Figure 3.**
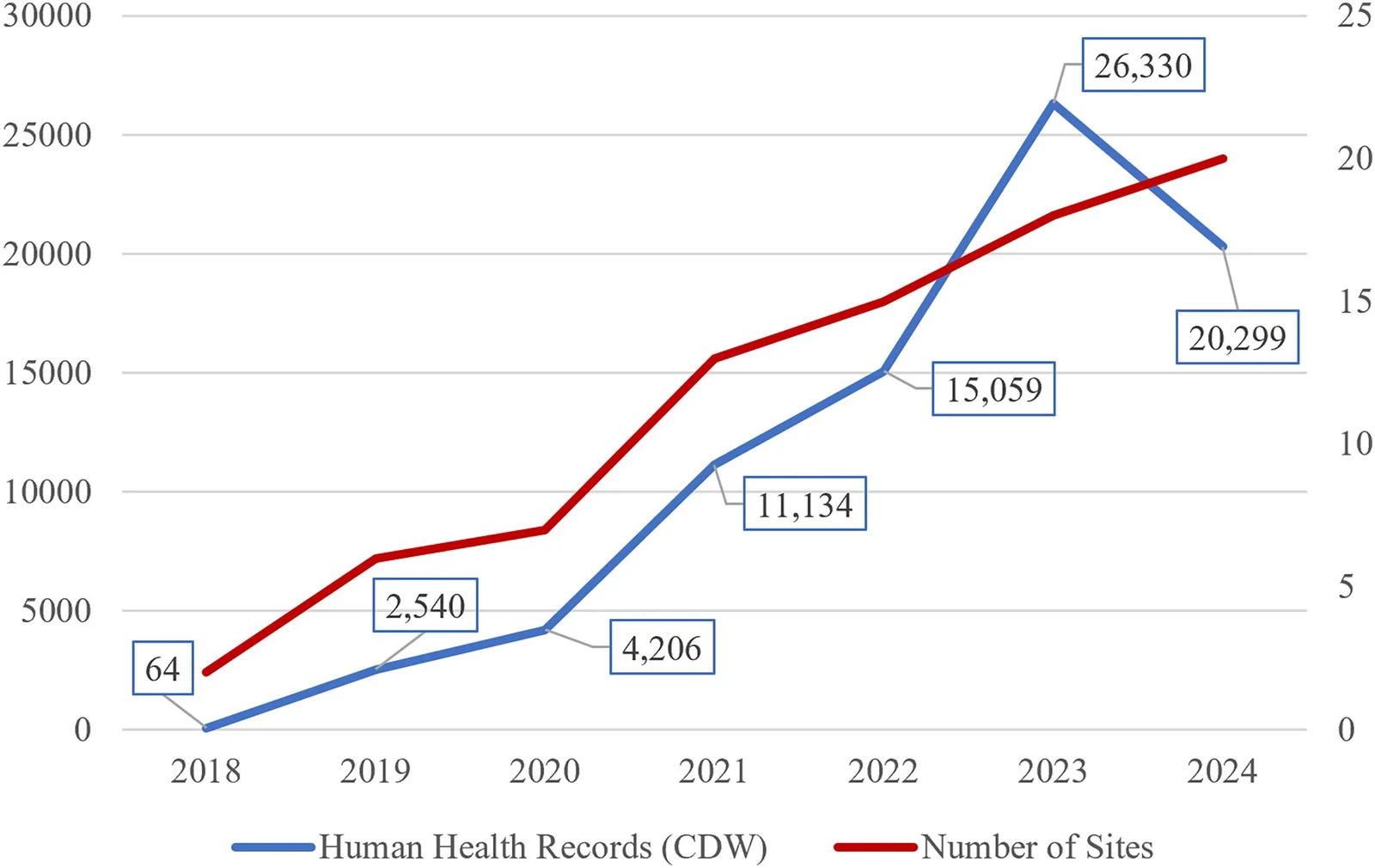
Human health antimicrobial resistance (AMR) surveillance data submitted to Central Data Warehouse (CDW), 2018–2024.

**Figure 4.**
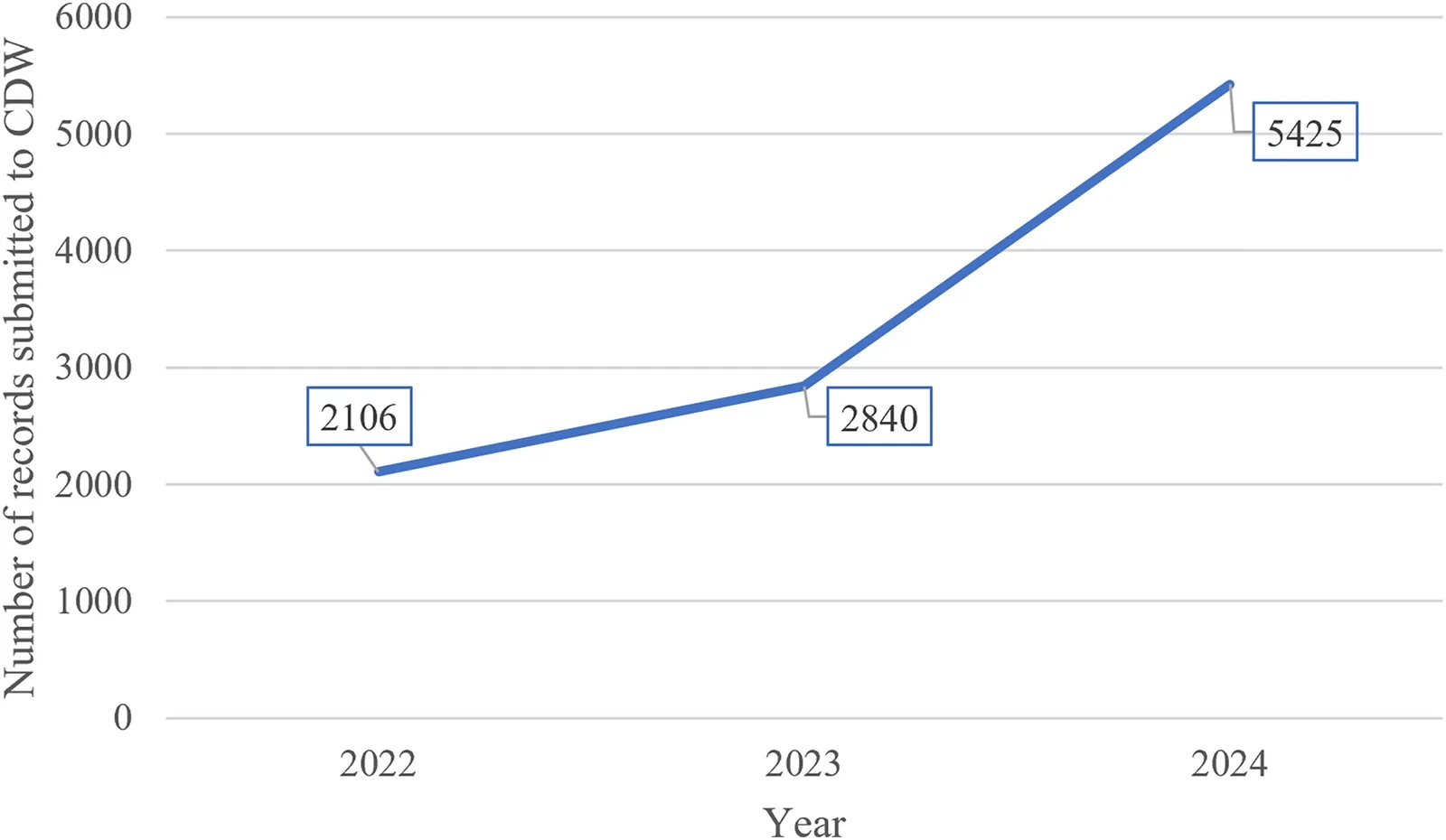
Animal health antimicrobial resistance (AMR) surveillance data submitted to Central Data Warehouse (CDW) 2022–2024.

Environmental AMR surveillance was introduced through a national sentinel site, completing the One Health picture across Figures 3–5. The Water Resources Authority was incorporated into the national surveillance network to initiate water-based AMR monitoring. The Central Water Testing Laboratory was renovated and equipped with instrumentation and reagents to undertake detection and resistance testing for environmental water samples. A standard operating procedure and protocol for environmental AMR surveillance were developed between 2024 and 2025, with pilot sampling generating 58 extended-spectrum beta-lactamase (ESBL)–producing *Escherichia coli* and *Klebsiella pneumoniae* samples by the end of 2024. Local studies have detected antibiotics in surface water at elevated concentrations, and an ongoing collaborative government-led project is assessing AMR transmission at the human–animal–environment interface [7]. However, environmental AMR surveillance remains in its early stages, with limited analytical infrastructure, narrow spatial and temporal coverage, and inadequate data for enhanced awareness and routine decision-making.

**Figure 5.**
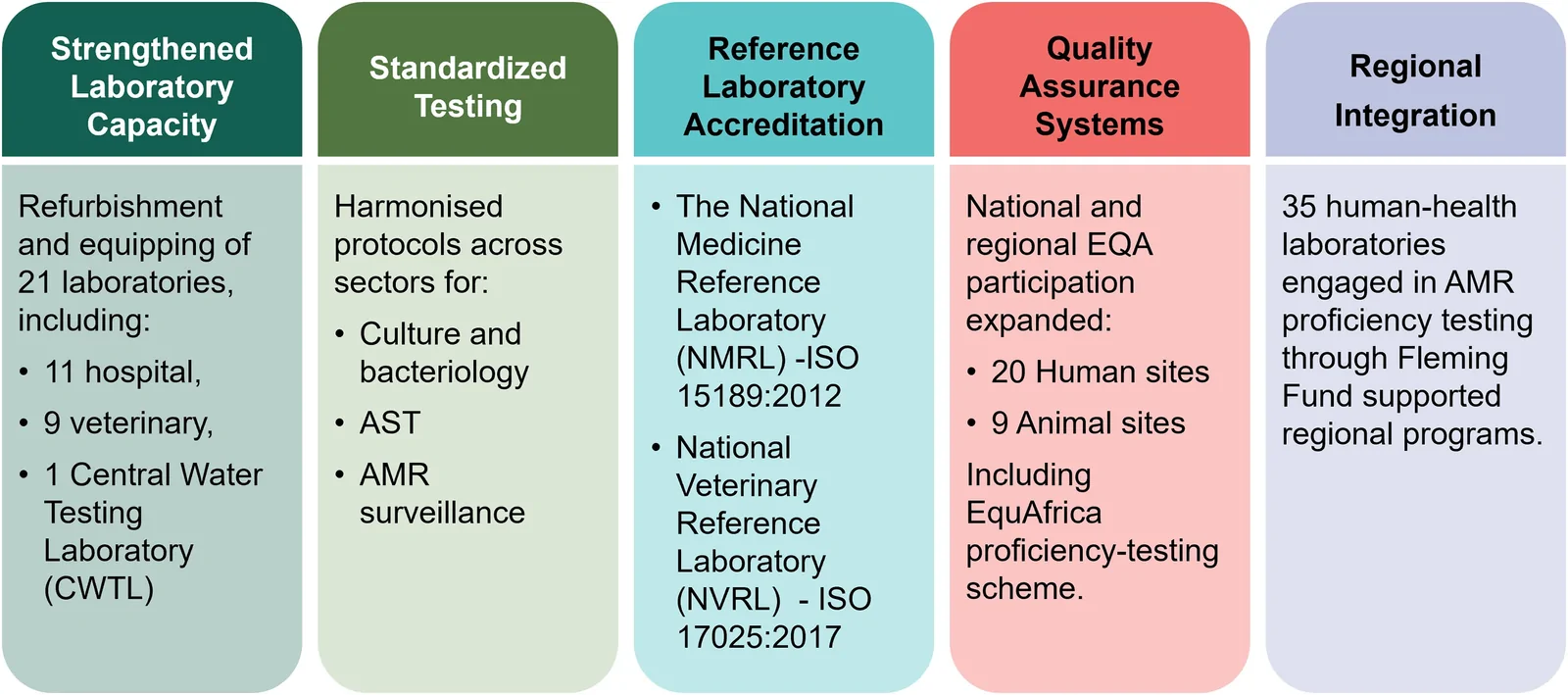
Laboratory systems strengthening supporting One Health antimicrobial resistance (AMR) surveillance in Kenya.

Laboratory quality performance improved across human and animal health sites with acceptable external quality assurance scores increasing from 13/26 (50%) to 23/28 (82%) [32], and reagent and consumable stock-outs falling from 14 in 2023 to 1 in 2025. These gains were supported by infrastructure, quality systems, and workforce capacity, as summarized in Figure 5 and Table 3.

**Table 3.** Workforce Capacity Development Across Human, Animal, and Environmental Health Sectors Supporting One Health AMR Surveillance in Kenya

Human Health	Animal Health	Environmental Health
42 professionals qualified as AMR Surveillance Experts across laboratory and epidemiology tracks, and over 1500 healthcare workers completed AMR-related training through the Ministry of Health (MoH) Virtual Academy. Structured mentorship and supervision visits further strengthened diagnostic stewardship and on-bench bacteriology competencies [7]. New capacities developed through SeqAfrica's training on bacterial Whole Genome Sequencing to facilitate future in-country testing.	26 professionals qualified as AMR Surveillance Experts through the Qualifying Workforce for AMR Surveillance (QWArS) program and Fleming Fund fellowship pathways. Veterinary laboratory scientists, epidemiologists, and surveillance officers also received training and mentorship in sampling, bacteriology, AMR data management, and laboratory quality systems.	10 technical officers from Water Resources Authority (WRA) and National Environment Management Authority (NEMA) were trained in environmental AMR surveillance, including sampling, sample handling, laboratory analysis, data management, and reporting, strengthening environmental integration within Kenya's One Health surveillance system.

Abbreviation: AMR, antimicrobial resistance.

### Data Production, Analysis, and Reporting

National data and reporting systems matured alongside facility expansion, with Kenya now reporting AMR and AMU data to WHO GLASS, WOAH ANIMUSE, and FAO InFARM, linking the country to global AMR data calls (Figure 6). National AMR reports evolved from early multisite summaries (2019–2021) to routine, multiyear, One Health national reports covering AMR, consumption and use. Under the National Action Plan on AMR 2.0 and the National One Health AMR Implementation Plan 2023–2027, outputs increasingly integrated AMC (from the Kenya Surveillance System for Antimicrobial Consumption system) [33], AMU (from hospital point prevalence surveys), and environmental AMR signals from the environmental sentinel site. Together, these efforts strengthened the analytical basis for stewardship, IPC/biosecurity, regulatory action, and broader One Health policy discussions.

**Figure 6.**
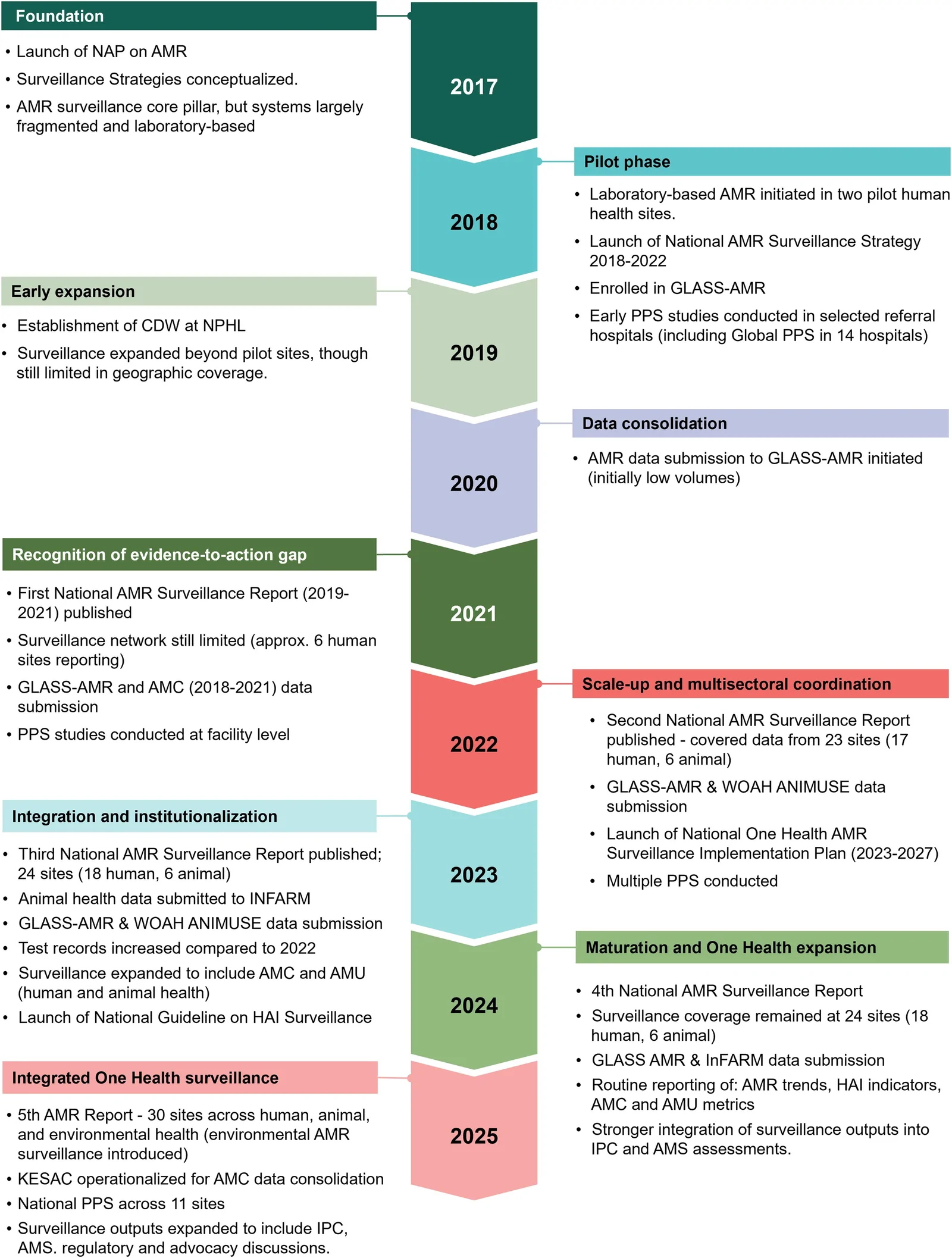
Surveillance milestones and evolution of antimicrobial resistance (AMR) surveillance in Kenya 2017–2025.

### Data Use for Action: Evidence-to-Decision Examples

Kenya's One Health AMR surveillance has increasingly shifted from data generation to supporting decision-making at clinical and programmatic levels through 3 main pathways: routine surveillance outputs, structured data quality review platforms, and decision products shaping clinical decisions, guidelines, stewardship, diagnostics, planning, and budgets.

Surveillance data contributed to revisions of the Kenya Essential Medicines List (KEML) [34], Essential Veterinary Medicines List [35], and Diagnostics List [36], and the development of national antibiotic treatment and surgical prophylaxis guidelines [37]. In human health, data guided more selective use of Watch and Reserve antibiotics and reduced reliance on broad-spectrum agents; in animal health, they informed prudent-use guidance for food-producing animals aligned with national priorities and WHO/WOAH classifications. The revised access watch and reserve (AWaRe)-aligned KEML informed the first Kenya National Medicines Formulary, linking prescribing norms and facility formularies to stewardship goals [38]. Surveillance evidence also informed Guidelines for Prudent Use of Antimicrobials across sectors [39]. These clinical reference tools supported treatment and stewardship decisions across human and animal health. AMR surveillance outputs were used to develop policy briefs, position statements, and presentation materials for national and county forums and partner dialogues, translating technical findings into accessible messages for advocacy, investment prioritization, and multisectoral alignment. Costed AMR workplans were incorporated into some County Integrated Development Plans, supporting county budget allocation for AMR interventions. Surveillance data also supported awareness among multiple disciplines and the public through facility and farm activities, professional associations, and annual World Antimicrobial Awareness Week events.

Beyond Kenya, these outputs supported regional dialogue and peer learning contributing to the development of Regional AMS Guidelines [40] and the African AMR Surveillance Guidance [41].

### Contribution Within a Multipartner Ecosystem

Kenya's AMR implementation has been enabled by a government-led, multipartner ecosystem coordinated through the multisectoral AMR Secretariat, NASIC, and CASICs. Within this landscape, the Fleming Fund strengthened the institutional and technical foundations of surveillance and microbiology across the human, animal, and environmental sectors, while complementary support from government agencies and partners, including public-private partnerships, contributed financing, training, advocacy, and subnational delivery [29]. National Antimicrobial Stewardship Interagency Committee also supported broader resource mobilization through multilateral and bilateral mechanisms aligned to One Health priorities and gaps identified during implementation. Stakeholder mapping in late 2023 confirmed broader multisectoral engagement but also highlighted gaps in underserved counties, reinforcing the need for partner alignment to NAP priorities and more equitable coverage.

The Fleming Fund-supported gains are best understood as catalytic contributions within a wider nationally coordinated AMR response, rather than outcomes attributable to any single actor.

### Impact on Joint External Evaluations and Tracking Antimicrobial Resistance Country Self-Assessment Survey

Between the 2017 and 2024 JEEs, Kenya demonstrated substantial progress in AMR-related capacities. In 2017 (JEE v1.0), AMR surveillance and AMS were assessed as “limited capacity” (level 2). By 2024 (JEE v3.0), more granular indicators showed “demonstrated capacity” (level 4) for AMR surveillance and multisectoral coordination, while AMS and multidrug-resistant organisms prevention reached “developed capacity” (level 3) [42, 43]. Tracking AMR Country Self-Assessment Survey responses indicated functional One Health governance, a costed surveillance strategy, and integrated reporting. While these benchmarks support the interpretation of meaningful progress, they reflect capacity assessments rather than direct outcome measures and should be interpreted alongside the operational data presented.

## DISCUSSION

Kenya's experience illustrates that sustainable AMR progress depends on treating AMR as a system-building agenda rather than a set of stand-alone, project-driven activities. From NAP-AMR 1.0 to the refinements in NAP-AMR 2.0 [12, 44], the country developed a clearer national–county governance architecture, with stronger operational roles for the AMR Secretariat, NASIC, and CASICs and greater emphasis on translating surveillance capacity into data use for decision-making and action. This trajectory mirrors wider global and regional shifts in AMR frameworks, from establishing AMR, AMU, and AMC surveillance systems to ensuring routine data are credible, comparable, and used for advocacy, education, stewardship, IPC/biosecurity, and policy.

External benchmarks support this interpretation. Between 2017 and 2024, Kenya moved from limited capacity to developed and demonstrated capacity for AMR-related JEE indicators.

### What Worked and Why

First, governance and coordination mechanisms matched devolution realities. Kenya's shift from advisory coordination to a clearer national–county One Health model demonstrates that, under devolution, AMR implementation works best when multisectoral structures are linked to county planning, budgeting, and reporting cycles and aligned with related structures such as IPC programs. By 2025, CASICs were present in 22 of 47 counties, and majority had budgeted workplans, suggesting that devolution can also support sustainability when counties align to NAPs.

Second, technical capacity mattered because it made surveillance data credible and usable for routine clinical and policy decisions. Investments in Quality Management Systems, sample handling, AST interpretation, and antibiogram development improved the quality and usefulness of AMR data for action.

Third, coordinated partner support added value when it reinforced a national plan rather than fragmenting it. Kenya's AMR program advanced through training, financing, advocacy, awareness, and IPC/biosecurity support, with strong government ownership and NASIC leadership enabling multisectoral progress across governance, surveillance, stewardship, and public engagement.

### Challenges and Constraints

1. Uneven subnational readiness and coverage: Devolution created variation in county capacity and prioritization, and where targeted support and resourcing were limited, coordination gains clustered in higher-capacity counties.
2. Animal health and environmental systems matured slower than human health: Early situational analyses showed fragmented laboratory capacity and surveillance in animal health sector, while environmental surveillance was absent at baseline. Current findings confirm that investment and progress in animal health and environmental systems remain slower than human health, particularly in laboratory expansion, routine international reporting, and stewardship-linked outputs. On-boarding the environmental sector into the surveillance system in particular remains constrained by limited awareness, analytical infrastructure, and narrow spatial and temporal coverage.
3. Multisectoral coordination remains hard to sustain: One Health coordination is institutionally demanding, requiring sustained financing, shared incentives, clear accountability, and time for relationship-building across ministries and departments. Where political commitment or leadership continuity is inconsistent, multisectoral mechanisms can stall without budget anchors and performance monitoring.
4. Translating data into routine action is still uneven: Even when surveillance improves, data-to-action requires consistent governance routines, stewardship and IPC/biosecurity capacity, robust supply chains, supervision, and enforcement. The remaining challenge is ensuring that One Health data trigger routine decisions across sectors, not only periodic reporting.

### Sustainability and Institutionalization

Kenya's NAP 2.0 reflects an intentional move toward sustainability by deliberately embedding AMR functions within institutions rather than individual projects [44]. Key measures include strengthening Secretariat capacity for M&E, Information Communication and Technology(ICT), and data management, formalizing national and county coordination structures, and advancing county work planning and resourcing. Beyond the Fleming Fund, sustainability will depend on 4 conditions:

1. Sustained and predictable domestic financing: One Health AMR activities incorporated into routine national and county planning and budgeting processes.
2. Institutionalized coordination: Stable coordination platforms backed by clear mandates, reporting arrangements, and continuity mechanisms.
3. Routine use of evidence: Data use, stewardship, and IPC/biosecurity oversight embedded in regular system practice rather than project-driven cycles.
4. Workforce capacity: The retention of trained AMR professionals across human, animal, and environmental sectors is an additional sustainability concern, though program documentation does not provide systematic data on workforce attrition or career trajectories beyond the training period during the project lifecycle.

Kenya's progress reflects substantial external catalytic investment, raising the question of what sustainability will realistically look like once donor support tapers. The likely answer is partial and phased sustainability, with core governance, laboratory testing, and reporting functions more readily absorbed into domestic budgets than higher-cost components such as advanced diagnostics, continuous mentorship, and comprehensive environmental surveillance, which may still require external support. This is likely not unique to Kenya but reflects a broader LMIC challenge of maintaining One Health AMR surveillance without widening inequities between higher-capacity and under-resourced settings.

### Study and Evidence Limitations

Study limitations include the inability to attribute outcomes to a single contributor given the multipartner ecosystem and potential gaps in routine data across counties and sectors. This is a narrative synthesis rather than a systematic review, and the findings reflect program documentation, secondary data, and stakeholder accounts rather than independent evaluation data.

### TRANSFERABLE IMPLEMENTATION PRINCIPLES

1. Design governance around administrative realities, not coordination ideals: Antimicrobial resistance governance is more effective when it matches how authority, planning, budgeting, and accountability actually work across national and subnational levels. In devolved settings, this means linking multisectoral coordination to existing county planning and budgeting cycles rather than creating parallel structures. For comparable settings, this implies institutionalizing end-to-end One Health coordination with clear national-subnational-facility reporting and feedback mechanisms and embedding AMR priorities within routine planning and financing at all levels.
2. Build credible laboratory and data systems before expecting routine data use: Strong surveillance depends on early investment in laboratory capacity, quality systems, standardization, and data management, across all sectors. Credibility of data is a prerequisite for trust, stewardship, and policy uptake. Strengthening surveillance data quality and establishing institutionalized data-to-action mechanisms should precede expectations of routine evidence-based decision-making.
3. Government-led, partner aligned approaches are critical: National AMR coordination mechanisms should set priorities, align partner contributions, and reduce duplication across donors and implementing partners. External investments are more durable when they reinforce existing national frameworks rather than operating alongside them. For sustainability, partner investments should be aligned to national action plan priorities across all sectors, prioritizing underserved counties and building durable coordination, data management, laboratory, and workforce capacities.
4. Integrate equity and data-to-action mechanisms from the onset: Early targeting of lower-capacity and under-resourced regions is essential to avoid widening geographic disparities. Embedding surveillance review meetings, IPC audits, stewardship rounds, and feedback loops into routine governance ensures that data inform practice, policy, and investment decisions across all sectors and regions. Maintaining human capacity, infrastructure, and supplies is essential for continuity of surveillance functions.

## CONCLUSION

Kenya's AMR journey spanning over a decade shows that durable progress is built incrementally through systems, not stand-alone projects. Within this trajectory, the Fleming Fund played a catalytic role in strengthening the technical and institutional foundations of One Health AMR surveillance across human, animal, and environmental sectors, helping translate national policy commitments into operational capacity and more credible data for stewardship, IPC, and policy action. The 2023–2027 NAP signals a further shift toward sustainability, with clearer roles for NASIC and CASICs, a stronger Secretariat, and greater emphasis on functionality, reporting, and resourcing. For Kenya and comparable settings, the lesson is that lasting AMR control depends on combining technical investment with governance financing and institutional routines that enable data to drive action at scale.
